# Comparative Effectiveness of Oral Hypoglycemic Agents for Glycemic Control and Glycemic Variability in Patients with Type 2 Diabetes Mellitus: Using Flash Glucose Monitoring

**DOI:** 10.2174/0115733998267817231227102553

**Published:** 2024-01-16

**Authors:** Poongothai Venkatachalapathy, Karthik Kumar Dos Alagarswamy Mohandoss, Murali Munisamy, Mohan Sellappan

**Affiliations:** 1 Department of Pharmacy Practice, Karpagam College of Pharmacy, Coimbatore, Tamil Nadu, India;; 2 Department of Obesity and Diabetes, GEM Hospital and Research Centre, Coimbatore, India;; 3 Department of Translational Medicine, All India Institute of Medical Sciences, Bhopal, Madhya Pradesh, India

**Keywords:** Glycemic control, glycemic variability, type 2 diabetes mellitus, oral hypoglycemic agents, flash glucose monitoring, ambulatory glucose profile

## Abstract

**Aim:**

The study aimed to compare the effectiveness of oral hypoglycemic agents (OHAs) as monotherapy, dual and quadruple therapy for glycemic control (GC) and glycemic variability (GV) in patients with type 2 diabetes (T2DM) using flash glucose monitoring system (FGM).

**Background:**

Diabetes management largely relies on HbA1c monitoring. Glycemic variability has been an evolving glycemic target for preventing complications related to type 2 diabetes mellitus.

**Objective:**

The purpose of the study was to compare glycemic control measures and glycemic variability measures among study groups and to study the relationships between GC and GV indices.

**Methods:**

Retrospectively, FGM data were collected from 50 T2DM patients. The patients were classified based on prescribed number of OHAs as monotherapy [group 1: Dipeptidyl peptidase- 4 (DPP-4) inhibitors (n=10), group 2: Sodium-glucose co-transporter-2 (SGLT2) inhibitors (n=10), group 3: Sulphonylureas (n=10), group 4: Dual therapy (n=10), and group 5: Quadruple therapy (n=10)]. Measures of GC and GV were evaluated.

**Results:**

Significant differences between study groups were observed in GC and GV measurements. The SGLT2 inhibitors monotherapy group demonstrated optimal GC [eA1c (%): 6.5 ± 2.2; MBG: 140.80 ± 63.94; TIR: 60.60 ± 19.96] and GV (SD: 42.38 ± 34.57; CV: 27.85 ± 6.68; MAGE: 96.76 ± 52.47; MODD: 33.96 ± 22.91) in comparison to other study groups. On using Pearson correlation analysis, mean blood glucose (MBG) and mean amplitude of glycemic excursion (MAGE) showed moderate correlation (r = 0.742)(r^2^ = 0.551), depicting distinct glucose variabilities at the same mean blood glucose levels.

**Conclusion:**

The monotherapy group of SGLT2 inhibitors demonstrated glucose-lowering effects with reduced glycemic variability. Hence, optimum glycemic control is associated with decreased glycemic variability.

## 1 INTRODUCTION

Current diabetes management largely relies on the ‘gold standard’ of glucose monitoring, *i.e*., HbA1c measurements. Patients with recurrent glycemic fluctuations, severe hyperglycemia, and hypoglycemic unawareness are recommended to use continuous glucose monitoring (CGM) by American Diabetes Association (ADA) guidelines 2019 [1], which has also been supported and published by Indian consensus guidelines on CGM 2019 [2]. American Association of Clinical Endocrinologists and American College of Endocrinology consensus statement 2019 suggests CGM for patients who cannot achieve glycemic target after 3 months of the initiation of oral hypoglycemic therapy [3].

Glycemic variability (GV) presenting with episodes of hypo and hyperglycemic excursions cannot be studied through HbA1c monitoring alone [4], where even two patients with the same HbA1c levels do not share similar GV patterns [5]. GV alone can act as an A1c-independent precipitating factor of micro and macrovascular complications, unpleasant cardiovascular outcomes, total mortality, and death [6]. A high GV has been demonstrated to be related to poor humanized health outcomes compared to HbA1c [7], and GV is affected by various oral hypoglycemic agents (OHAs) [8]. The glycemic excursions are reduced by drugs, like acarbose, which potentially concentrate on post-prandial hyperglycemia [9]. Controlled-release preparation of glipizide was found to reduce the mean amplitude of glycemic excursions (MAGE) in combination with acarbose than alone [10]. Newer treatment modalities in diabetes, like GLP- 1 analogues, DPP-4 inhibitors, and bariatric surgical procedures, adress the topic of GV efficiently, and also investigating GV with the use of such drugs is promising in enhanced diabetes management [11]. Although the effects of OHAs on glycemic control (GC) have been well studied and documented in large clinical studies, their effect on GV is not well established. The utilization of advanced CGM techniques to measure GC and GV indices in both T1DM and T2DM patients for personalizing diabetes care is a promising futuristic goal in the precision medicine of diabetes [6].

The current study was carried out to compare the effects of OHAs as monotherapy, dual therapy, and quadruple therapy on glycemic control and glycemic variability in T2DM patients using flash glucose monitoring (FGM) along with ambulatory glucose profile (AGP) reports.

## 2 MATERIALS AND METHODS

### 2.1 Study Design and Patients

This retrospective cohort study involving outpatients with T2DM was performed using medical records from two centers, Gastroenterology Medical Centre and Hospital (GEM) and Karpagam Faculty of Medical Sciences and Research (KFMSR), Coimbatore. Ethical approval was obtained from the institutional ethics committee KFMSR (IEC number: IHEC/204/KCOP/2020).

Inclusion criteria were adults aged 18–80 years with T2DM for > 6 months, patients with AGP data, and those receiving OHAs. We excluded patients with the following conditions: (1) type 1 diabetes; (2) gestational diabetes mellitus; (3) pregnant or lactating women; (4) diabetes due to secondary causes (*e.g.,* chronic pancreatitis, pancreatic cancer, infection-related diabetes, steroid-induced diabetes mellitus); (5) end-stage renal failure requiring dialysis; and (6) active anticancer therapy. Selected patients were then grouped based on the number of prescribed OHAs prior to FGM monitoring as monotherapy [group 1: Dipeptidyl peptidase- 4 (DPP-4) inhibitors, group 2: Sodium-glucose co-transporter-2 (SGLT2) inhibitors, group 3: Sulphonylureas], group 4: Dual therapy (sulphonylureas + dipeptidyl peptidase-4 inhibitors), and group 5: Quadruple therapy (sulphonylureas + dipeptidyl peptidase-4 inhibitors + sodium-glucose cotransporter-2 inhibitors along with metformin).

Although metformin is the first drug of choice for diabetes, FGM monitoring is not advised at such an initial stage; hence, we were not able to include the metformin monotherapy group. As GV can be analyzed using CGM techniques, like the FGM system, only fewer subsets of patients with diabetes having a clinical rationale for such application are advised to be monitored by using real-time glucose monitoring devices. Because of this, the study has a small sample size. The flow chart of the study is summarized in Fig. (**1**).

### 2.2 Study Procedure

An electronic medical records dataset from November 2019 to August 2020 (9 months) was accessed to collect data on all eligible patients with T2DM. The baseline characteristics of patients, such as age, sex, co-morbidities, HbA1c levels, and anti-diabetic medication prescribed, were collected. An ambulatory glucose profile report obtained using Free Style Libre-Pro (FSLP) (Libre Pro, Abbott Laboratories, Lake Bluff, Illinois) flash glucose monitoring device was utilized in this study. This device was attached to the patient’s upper arm by the physician. The device is comprised of 3 components- the sensor, the reader, and the interpretative software. Once applied, the sensor has a 2 weeks shelf-life and measures interstitial glucose level every 15 minutes for 14 days. It provides up to 1340 glucose measurements and the data are stored on a memory chip inside the sensor disc. The sensors were removed after 14 days of application by the physician. The glucose data captured by the sensor were retrieved by flashing the reader against the sensor and downloaded to the computer storage. The inbuilt software then condensed the data and provided an automated and standardized visualization of the numerical and graphic display of glycemic patterns called the ambulatory glucose profile report.

### 2.3 Measures of Glycemic Control (GC)

The glycemic control measures obtained from the AGP report included estimated A1c (eA1c) in percentage and mmol/mol, mean blood glucose (MBG), time in target range (TIR), time below range (TBR), and time above range (TAR).

### 2.4 Measures of Glycemic Variability (GV)

The raw FGM data file was used to calculate glycemic variability indices using a web application ‘GlyCulator-2.0’ [12], which included standard deviation (SD), coefficient of variation (CV), mean amplitude of glycemic excursion (MAGE), mean of daily difference (MODD), continuous overlapping net glycemic action (CONGA), low blood glucose index (LBGI), high blood glucose index (HBGI), M-value, J-index, GRADE, % GRADE (hypoglycemia), % GRADE (euglycemia), and % GRADE (hyperglycemia).

### 2.5 Statistical Analysis

Statistical analyses were performed using Statistical Package for Social Sciences (SPSS) software, version 21.0. Data are presented as means ± SD, unless otherwise specified. Patient characteristics, such as sex and comorbidities, are presented as n (%). Two-tailed paired t-test was utilized for testing the changes from baseline HbA1c (%) to eA1c (%). One-way ANOVA was used to compare glycemic control (GC) measures and glycemic variability (GV) measures among the study groups. The relationships between GC and GV indices were studied using Pearson’s correlation analysis.

## 3 RESULTS

### 3.1 Baseline Patient Characteristics

**Fig. (1):**
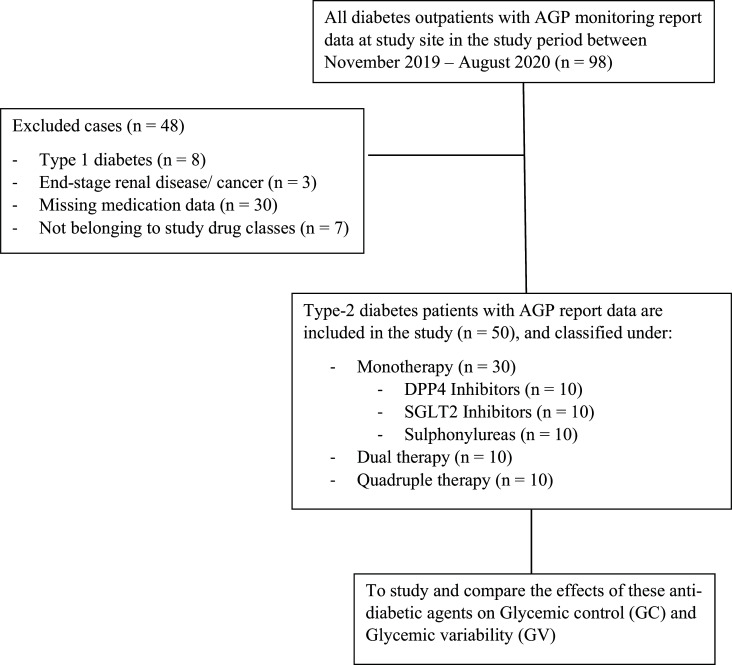
The flowchart of the study. AGP: Ambulatory glucose profile.

A total of 50 patients met the inclusion criteria and were categorized based on the number of medications prescribed into respective study groups with 10 patients in each group (Fig. **1**). Among 50 patients, 35 (70%) were male and 15 (30%) were female, with a mean age of 52.76 ±11.03. HbA1c (%) measured within the 3 months before the application of FSLP- FGM was considered as baseline. The mean baseline HbA1c (%) was found to be 9.4 ± 3.2 in the DPP-4 inhibitors group, 8.2 ± 2.4 in the SGLT2 inhibitors group, 10.8 ± 2.1 in sulphonylureas group, 9.4 ± 1.3 in dual therapy group, and 11.7 ± 2.4 in quadruple therapy group. Among these, the quadruple therapy group had the highest mean baseline HbA1c value (%) of about 11.7 ± 2.4. The patients had been medically diagnosed with concurrent illnesses of hypertension [n=22 (44%)], hyperlipidemia [n=19 (38%)], and hypothyroidism [n=2 (4%)] (Table **1**).

### 3.2 Glycemic Control (GC)

As compared to the baseline, statistically significant reductions were observed between baseline HbA1c (%) and eA1c (%) levels with DPP4 Inhibitors (*p =* 0.0175), SGLT2 inhibitors (*p =* 0.0071), and quadruple therapy groups (*p =* 0.0052). Whereas, sulphonylureas and dual therapy groups showed insignificant reductions (*p*>0.05). There were significant differences in eA1c (%) (*p =* 0.03820), eA1c (mmol/mol) (*p =* 0.03846), MBG (*p =* 0.03835), TIR (*p =* 0.00017), and TAR (*p =* 0.00050) levels found between antidiabetic monotherapy, dual and quadruple therapy. The glycemic control measures [eA1c (%), eA1c (mmol/mol), MBG, TIR, TAR, TBR] were studied to be 8.2 ± 3.7, 66.60 ± 41.18, 190.20 ± 108.44, 35.90 ± 26.55, 54.60 ± 32.90, and 9.50 ± 8.94 in DPP-4 inhibitors monotherapy group; 6.5 ± 2.2, 47.80 ± 24.33, 140.80 ± 63.94, 60.60 ± 19.96, 30.00 ± 22.80, and 9.40 ± 8.01 in SGLT2 inhibitors monotherapy group; 10.4 ± 2.8, 91.00 ± 31.53, 254.30 ± 82.72, 14.00 ± 16.63, 81.70 ± 23.45, and 4.30 ± 6.88 in sulphonylureas group; 7.9 ± 2.2, 63.30 ± 24.34, 181.100 ± 63.34, 29.60 ± 21.61, 64.30 ± 28.97, and 6.10 ± 11.95 in the dual therapy group, and 9.1 ± 2.4, 76.10 ± 27.12, 214.80 ± 71.43, 22.70 ± 18.98, 75.80 ± 19.65, and 1.50 ± 2.71 in the quadruple therapy group (Table **2**). There were no significant differences in TBR demonstrated between various drug categories (*p =* 0.16410). The periods of TBR were comparatively lower than TAR in all drug groups. However, lower incidences of TBR were demonstrated in the quadruple therapy group (1.50 ± 2.71) and higher incidences of TBR in the DPP-4 inhibitors group (9.50 ± 8.94).

### 3.3 Glycemic Variability (GV)

**Fig. (2):**
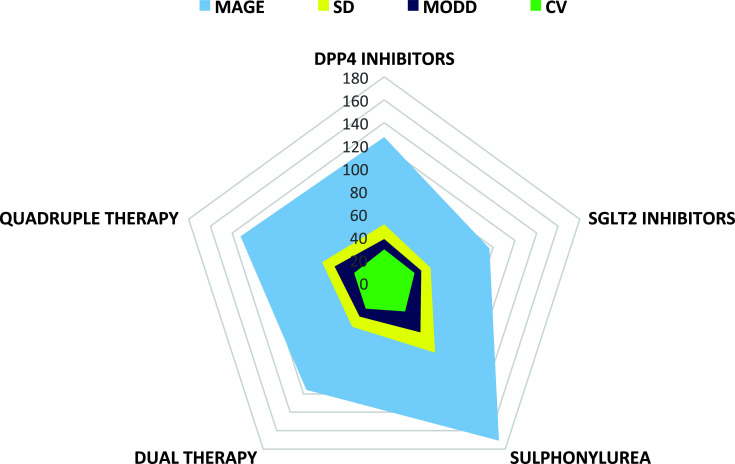
Comparative graphical representation of glycemic variability (GV) measures, including MAGE, SD, MODD, and CV. DPP-4, Dipeptidyl peptidase-4; SGLT2, Sodium-glucose co-transporter-2; MAGE, Mean amplitude of glycemic excursions; SD, Standard deviation; MODD, Mean of daily difference; CV, Coefficient of variation.

There were statistically significant differences in SD (*p =* 0.024493), MAGE (*p =* 0.002332), M100 (*p =* 0.013432), GRADE (*p =* 0.025199), % GRADE (euglycemia) (*p =* 0.000032), and % GRADE (hyperglycemia) (*p =* 0.001772) among the study groups. However, there were no significant differences in CV, MODD, LBGI, HBGI, CONGA1h, J index, and GRADE (hypoglycemia) between the groups (*p*>0.05). On using the one-way ANOVA test, glycemic variability measures (SD, CV, MAGE, MODD, HBGI, M100, J index, GRADE, % GRADE (euglycemia), and % GRADE (hyperglycemia)] were found to be lower in SGLT2 inhibitors monotherapy group (42.38 ± 34.57, 27.85 ± 6.68, 96.76 ± 52.47, 33.96 ± 22.91, 5.49 ± 12.29, 146.75 ± 115.92, 42.29 ± 59.51, 6.12 ± 7.35, 28.44 ± 14.06, and 61.01 ± 20.23) and higher in sulphonylureas group (75.48 ± 25.08, 31.06 ± 8.80, 170.63 ± 41.76, 53.65 ± 23.18, 26.06 ± 16.20, 375.95 ± 137.38, 117.26 ± 59.69, 19.83 ± 9.04, 3.36 ± 5.75 and 92.49 ± 14.42), respectively (Table **3** and Fig. **2**). LBGI and GRADE (hypoglycemia) were studied to be lower in the quadruple therapy group (0.25 ± 0.41, 0.77 ± 1.73) and higher in SGLT2 inhibitors monotherapy group (1.17 ± 1.08, 10.54 ± 16.68). The quadruple therapy group and sulphonylureas group showed the lowest and highest CONGA (1h) levels of 19.32 ± 3.88 and 25.15 ± 8.47, respectively.

### 3.4 Relationship between Glycemic Control (GC) and Glycemic Variability (GV) Measures

**Fig. (3):**
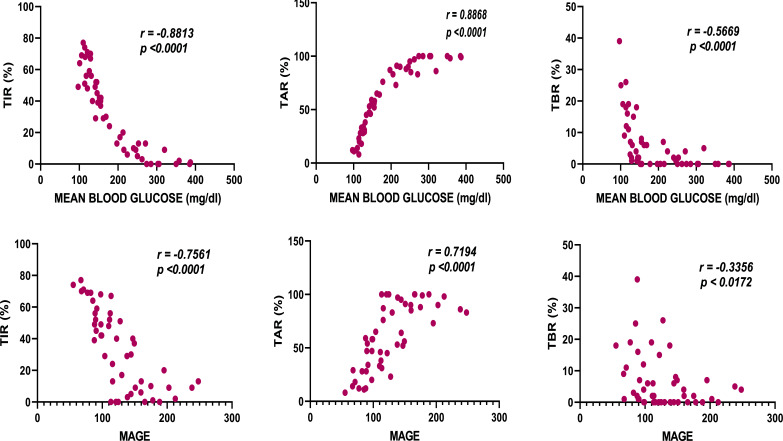
Correlation among glycemic control (GC) and glycemic variability (GV) indices. TIR, Time in range; TAR, Time above range; TBR, Time below range; MAGE, Mean amplitude of glycemic excursion.

Correlation tests were performed on GC and GV measures of all groups collectively using two-tailed Pearson correlation tests. A positive association was observed between MBG – TAR (r= 0.8868) and MAGE – TAR (r= 0.7194), which has been found significant at the p-value of 0.01, indicating a linear relationship (Fig. **3**). A strong negative association was observed between MBG - TIR (r= - 0.8813) and MAGE - TIR (r = - 0.7561), showing the inverse relationship. A negative relationship was observed between MBG – TBR (r= - 0.5669) and MAGE – TBR (r = - 0.3356) with moderate and weak strengths of the association, respectively.

All study groups showed moderate to very strong correlation between MBG and MAGE with r-values of 0.778 in the DPP-4 inhibitors group (very strong), 0.956 in the SGLT-2 inhibitors group (very strong), 0.597 in the sulphonylureas group (moderate), 0.557 in the dual therapy group (moderate), and 0.510 in the quadruple therapy group (moderate). However, moderate correlation was observed when all groups were collectively accounted for (r = 0.742, r^2^ = 0.551; *p*>0.001), depicting distinct glucose variabilities at the same mean blood glucose levels.

## 4 DISCUSSION

In the current study, we have analyzed the effects of OHAs as monotherapy, dual therapy, and quadruple therapy on GC and GV amongst individuals with T2DM. We have observed significant differences in the effects of OHAs on GC and GV indices using the FGM system. Newer CGM technologies play a significant role in precise diabetes management through personalized investigation of glucose profiles in patients with diabetes. The Diabetes Control and Complications Trial (DCCT) group has established that HbA1c variations influence the progression of microvascular complications, like retinopathy and nephropathy [13]. Statistically significant reductions were observed between baseline HbA1c (%) and eA1c (%) in DPP4 inhibitors, SGLT2 inhibitors, and quadruple therapy groups, respectively. Among these, SGLT2 Inhibitors exhibited glycemic regulation with the lowest reported mean eA1c (%) values, which have been found to be consistent with previously reported clinical studies [14].

Various clinical trials have established the association between GC and the prevention of diabetic complications. There have been significant differences found in eA1c (%), eA1c (mmol/mol), MBG, TIR, and TAR between the study groups. Patients receiving SGLT2 inhibitors and sulphonylureas monotherapy presented with the lowest and highest eA1c (%), eA1c (mmol/ml), MBG, and TAR, respectively, in comparison to the other groups. This is consistent with phase 2 and 3 clinical trials on SGLT-2 inhibitors, demonstrating improved glycemic control as monotherapy in patients with T2DM [15]. An association between TIR and the risk of development of diabetic retinopathy (DR), microalbuminuria, and other microvascular complications, has been studied [16]. Increasing time in range (TIR) while reducing time below range (TBR) and time above range (TAR) is the principal goal in glycemic optimization [17]. All the drug groups showed reduced TBR in comparison to TAR. Hypoglycemia is generally known to be less in patients with T2DM than in patients with T1DM; hence, desired time in target range can be attained by reducing hypoglycemic events. A higher percentage of TIR with a lower percentage of TAR was observed in patients receiving SGLT-2 Inhibitors. In a pilot study on Japanese type 1 diabetes patients, SGLT2 inhibitors were studied to enhance TIR without increasing TBR [18]. Whereas, sulphonylureas exhibited extensive hyperglycemic periods signified by the TAR percentage of 81.70 ± 23.45 with a lessened TIR percentage of 14.00 ± 16.63.

Glycemic variability is an integral unit of glucose homeostasis. GV gradually increases from ‘prediabetes through T2DM evolution’. A dependable marker of oxidative stress, 8-iso-PGF2α, with its urinary excretion rate was established to be positively correlated with GV [19]. Given the significance, GV is developing as an additional significant glycemic target. Most commonly used GV metrics include % coefficient of variation (% CV) and standard deviation (SD). SD, CV, MAGE, and CONGA(1h) are used to measure intraday glycemic variability, in which, MAGE remains the comprehensive index for evaluating intraday variability [20]. There were significant differences observed in SD, MAGE, M100, GRADE, % GRADE (euglycemia), and % GRADE (hyperglycemia) between monotherapy, dual, and quadruple therapy groups. Patients belonging to the SGLT2 inhibitors and sulphonylureas monotherapy groups showed reduced and elevated GV with lowest and highest SD, MAGE, M100, GRADE, and % GRADE (hyperglycemia), respectively, in comparison to the other groups. Some earlier studies have also demonstrated the association between the use of sulphonylureas and increased GV [21], which was also confirmed in a recent study conducted on classifying sulphonylureas as GV-increasing medications [22]. However, MODD remains the sole parameter for measuring interday glycemic variability. Although, there were no significant differences observed in MODD between drug groups, the lowest MODD was reported in the SGLT2 inhibitors group, signifying that SGLT2 inhibitors improve both intra and interday glucose variability. This is consistent with a recent study on SGLT2 inhibitors in type 1 diabetes patients [23]. Next to SGLT-2 inhibitors, the dual therapy group also showed reduced GV indices of SD, MAGE, and MODD, in comparison to other groups.

LBGI and HBGI are common indices employed to compute the risk of hypo- and hyperglycemia. There were no statistically significant differences in LBGI and HBGI observed between the drug therapy groups. The SGLT-2 inhibitors group showed increased LBGI and decreased HBGI. The sulphonylureas group exhibited elevated HBGI of 26.06 ± 16.20. M100 value and the J index have been studied as indices of the quality of glycemic control in an effort to amalgamate both the mean glucose level and GV in a single unit. M100 and J-index values have been found to be high in the sulphonylureas group (375.95 ± 137.38; 117.26 ± 59.69) and low in the SGLT2 inhibitors group (146.75 ± 115.92, 42.29 ± 59.51). Glycemic Risk Assessment Diabetes Equation (GRADE) is used to assess clinical risk scores using glycemic patterns. There were significant differences in GRADE, % GRADE (euglycemia), and % GRADE (hyperglycemia) between the groups. The SGLT2 inhibitors group displayed better (GRADE), % GRADE (euglycemia), and % GRADE (hyperglycemia) values in comparison to other groups.

Consistent with the outcomes of the ADAG study [24], we found a perfect correlation between eA1c (%) and MBG levels (mg/dl) (r = 0.99). TAR was studied to increase with an increase in MBG and MAGE. Whereas, TIR was found to decrease with an increase in MBG (r = - 0.8813) and MAGE (r = -0.7561). This finding is in accordance with the results of a recent study, involving both type 1 and type 2 diabetes patients, in which a good negative correlation was observed between HbA1c and TIR (r= -0.84, r^2^=0.71) [25]. However, TBR showed moderate and weak negative associations with MBG and MAGE, respectively. The relationship between GC and GV was studied by analysing the correlation between MBG and MAGE. In consensus with findings from the ADAG study [26], MBG showed a moderate correlation with MAGE (r = 0.742) (r^2^ = 0.551). This demonstrates that patients even with the same mean blood glucose and A1c range experience varied intra and inter-glycemic variability.

Overall, the SGLT2 inhibitors monotherapy group demonstrated glycemic regulation effects with decreased glycemic fluctuations, as revealed by lower levels of GV measures. In a meta-analysis on SGLT2 inhibitors in 2013, the glucose-reducing efficiency was found to be on average 0.79% with monotherapy and 0.61% when used in combination in patients with baseline HbA1c levels of 6.9-9.2% [27]. Also, positive pleiotropic outcomes of SGLT2 inhibitors on body weight, systolic blood pressure, and estimated glomerular filtration rate values with enhanced cardiovascular outcomes, have been established by various clinical trials [28]. As a higher number of antidiabetic agents have been demonstrated to have no association with optimal GC and GV, the effects of medications on GC and GV should be given importance [29]. In the SGLT-2 inhibitors group with the lowest mean eA1c (%) of 6.5 ± 2.2, significantly lower levels of intra and inter-glycemic variation were observed. This finding is consistent with the results of an observational study on the Caucasian population, which demonstrated that reduced GV is associated with better GC [30]. The limitations of our study were the small sample size and the sulphonylureas drug, glibenclamide, not being included in our study. As a future direction, a long-term prospective cohort study can be designed to corroborate our findings.

## CONCLUSION

In conclusion, we have compared retrospectively the effects of OHAs as monotherapy, dual, and quadruple therapies on GC and GV in patients with T2DM using FGM. GC and GV measures varied significantly between the study groups. Among all groups, the SGLT-2 inhibitors monotherapy group had exhibited glucose-lowering outcomes with reduced glycemic excursions. Whereas the sulphonylureas group displayed high GV with extended hyperglycemic periods. Hence, optimum GC is associated with reduced GV.

## Figures and Tables

**Table 1:** Baseline patient characteristics (n=50).

**Baseline Characteristics**	**Monotherapy**			**Dual Therapy (n=10)**	**Quadruple Therapy (n=10)**
	**DPP4 Inhibitors (n=10)**	**SGLT2 Inhibitors (n=10)**	**Sulphonylurea (n=10)**		
	**Mean ± SD or n (%)**				
Male	7 (70%)	8 (80%)	10 (100%)	5 (50%)	5(50%)
Female	3 (30%)	2 (20%)	0 (0%)	5 (50%)	5 (50%)
Age	59.20 ± 12.24	51.00 ± 9.16	49.60 ± 11.04	52.30 ± 10.56	51.70 ± 11.54
HbA1c	9.4 ± 3.2	8.2 ± 2.4	10.8 ± 2.1	9.4 ± 1.3	11.7 ± 2.4
Hypertension	5 (50%)	2 (20%)	3 (30%)	6 (60%)	6 (60%)
Hyperlipidemia	4 (40%)	2 (20%)	2 (20%)	5 (50%)	6 (60%)
Hypothyroidism	0 (0%)	1 (10%)	0 (0%)	0 (0%)	1 (10%)

**Note:** Data are presented as mean ± SD, n (%). DPP-4: Dipeptidyl peptidase-4; SGLT2: Sodium glucose cotransporter-2; HbA1c: Hemoglobin A1c.

**Table 2:** Glycemic control.

**Measures**	**Monotherapy**				**Dual Therapy (n=10)**	**Quadruple Therapy (n=10)**	**p-value**
	**DPP4 Inhibitors (n=10)**		**SGLT2 Inhibitors (n=10)**	**Sulphonylurea (n=10)**			
	**Mean ± SD**						
eA1c (%)	8.2 ± 3.7	6.5 ± 2.2		10.4 ± 2.8	7.9 ± 2.2	9.1 ± 2.4	0.03820*
eA1c (mmol/mol)	66.60 ± 41.18	47.80 ± 24.33		91.00 ± 31.53	63.30 ± 24.34	76.10 ± 27.12	0.03846*
Mean blood glucose (mg/dl)	190.20 ± 108.44	140.80 ± 63.94		254.30 ± 82.72	181.100 ± 63.34	214.80 ± 71.43	0.03835*
Time in range (TIR)	35.90 ± 26.55	60.60 ± 19.96		14.00 ± 16.63	29.60 ± 21.61	22.70 ± 18.98	0.00017*
Time below range (TBR)	9.50 ± 8.94	9.40 ± 8.01		4.30 ± 6.88	6.10 ± 11.95	1.50 ± 2.71	0.16410
Time above range (TAR)	54.60 ± 32.90	30.00 ± 22.80		81.70 ± 23.45	64.30 ± 28.97	75.80 ± 19.65	0.00050*

**Note:** Data are presented as mean ± SD. Correlation of glycemic control measures between oral hypoglycemic agents’ monotherapy, dual therapy, and quadruple therapy using one-way ANOVA. *p<0.05 was considered significant. DPP-4: Dipeptidyl peptidase-4; SGLT2: Sodium glucose cotransporter-2; eA1c: Estimated A1c.

**Table 3:** Glycemic variability.

**Measure**	**Monotherapy**				**Dual Therapy (n=10)**	**Quadruple Therapy (n=10)**	***p*-value**
	**DPP4 Inhibitors (n=10)**	**SGLT2 Inhibitors (n=10)**		**Sulphonylurea (n=10)**			
	**Mean ± SD**						
SD	51.13 ± 19.21		42.38 ± 34.57	75.48 ± 25.08	47.42 ± 10.77	57.33 ± 18.32	0.024493*
CV	29.24 ± 7.35		27.85 ± 6.68	31.06 ± 8.80	27.93 ± 7.75	27.84 ± 8.35	0.860870
MAGE	127.22 ± 36.36		96.76 ± 52.47	170.63 ± 41.76	115.83 ± 23.58	132.05 ± 34.44	0.002332*
MODD	38.08 ± 14.56		33.96 ± 22.91	53.65 ± 23.18	36.70 ± 9.92	45.63 ± 11.73	0.094196
CONGA(1h)	23.49 ± 4.96		20.82 ± 5.18	25.15 ± 8.47	20.92 ± 3.25	19.32 ± 3.88	0.139866
LBGI	1.10 ± 1.02		1.17 ± 1.08	0.59 ± 0.95	0.91 ± 1.92	0.25 ± 0.41	0.395275
HBGI	14.75 ± 21.18		5.49 ± 12.29	26.06 ± 16.20	11.25 ± 11.17	17.18 ± 14.41	0.061880
M100	250.10 ± 185.73		146.75 ± 115.92	375.95 ± 137.38	247.25 ± 119.38	301.73 ± 135.08	0.013432*
J index	72.42 ± 77.57		42.29 ± 59.51	117.26 ± 59.69	56.61 ± 34.95	80.01 ± 49.45	0.064306
GRADE	12.07 ± 12.11		6.12 ± 7.35	19.83 ± 9.04	11.49 ± 7.27	14.98 ± 8.61	0.025199*
% GRADE (hypoglycemia)	7.84 ± 8.78		10.54 ± 16.68	4.14 ± 8.82	7.82 ± 21.03	0.77 ± 1.73	0.517200
% GRADE (euglycemia)	14.41 ± 14.61		28.44 ± 14.06	3.36 ± 5.75	9.20 ± 8.01	6.78 ± 7.37	0.000032*
% GRADE (hyperglycemia)	77.73 ± 20.93		61.01 ± 20.23	92.49 ± 14.42	82.97 ± 23.40	92.43 ± 7.29	0.001772*

**Note:** Data are presented as mean ± SD. Correlation of glycemic variability measures between oral hypoglycemic agents’ monotherapy, dual therapy, and quadruple therapy using one-way ANOVA. * *p*<0.05 was considered significant. DPP-4: Dipeptidyl peptidase-4; SGLT2: Sodium glucose cotransporter-2.

## Data Availability

The data and supportive information are available within the article.

## LIST OF ABBREVIATIONS

DCCT: Diabetes Control and Complications Trial
GRADE: Glycemic Risk Assessment Diabetes Equation
SD: Standard Deviation
TAR: Time Above Range
TBR: Time Below Range
TIR: Time in Range

## STANDARDS OF REPORTING

STROBE guidelines were followed.

## References

[1] International Diabetes Federation (2019). IDF Diabetes Atlas..

[2] Saboo B., Chawla M., Jha S. (2019). Consensus and recommendations on continuous glucose monitoring.. J. Diabetology.

[3] Garber A.J., Abrahamson M.J., Barzilay J.I. (2019). Consensus statement by the American association of clinical endocrinologists and American college of endocrinology on the comprehensive type 2 diabetes management algorithm – 2019 executive summary.. Endocr. Pract..

[4] Chehregosha H., Khamseh M.E., Malek M., Hosseinpanah F., Ismail-Beigi F. (2019). A view beyond HbA1c: Role of continuous glucose monitoring.. Diabetes Ther..

[5] Kohnert K-D., Vogt L., Salzseider E. (2010). Advances in understanding glucose variability and the role of continuous glucose monitoring.. Eur. J. Endocrinol..

[6] Ceriello A., Monnier L., Owens D. (2019). Glycaemic variability in diabetes: Clinical and therapeutic implications.. Lancet Diabetes Endocrinol..

[7] Penckofer S., Quinn L., Byrn M., Ferrans C., Miller M., Strange P. (2012). Does glycemic variability impact mood and quality of life?. Diabetes Technol. Ther..

[8] Unnikrishnan A.G., Purandare V.B. (2020). Ambulatory glucose profile as an educational tool in the management of patients with type 2 diabetes mellitus.. IJMRHS.

[9] Li Y., Xu L., Shen J. (2010). Effects of short-term therapy with different insulin secretagogues on glucose metabolism, lipid parameters and oxidative stress in newly diagnosed Type 2 Diabetes Mellitus.. Diabetes Res. Clin. Pract..

[10] Bao Y.Q., Zhou J., Zhou M. (2010). Glipizide controlled‐release tablets, with or without acarbose, improve glycaemic variability in newly diagnosed Type 2 diabetes.. Clin. Exp. Pharmacol. Physiol..

[11] Suh S., Kim J.H. (2015). Glycemic variability: How do we measure it and why is it important?. Diabetes Metab. J..

[12] Pagacz K., Stawiski K., Szadkowska A., Mlynarski W., Fendler W. (2018). GlyCulator2: An update on a web application for calculation of glycemic variability indices.. Acta Diabetol..

[13] The DCCT Research Group (1986). The Diabetes Control and Complications Trial (DCCT). Design and methodologic considerations for the feasibility phase.. Diabetes.

[14] Hsia D.S., Grove O., Cefalu W.T. (2017). An update on SGLT2 inhibitors for the treatment of diabetes mellitus.. Curr. Opin. Endocrinol. Diabetes Obes..

[15] Chao E.C. (2014). SGLT-2 inhibitors: A new mechanism for glycemic control.. Clin. Diabetes.

[16] Hirsch I.B., Sherr J.L., Hood K.K. (2019). Connecting the dots: Validation of time in range metrics with microvascular outcomes.. Diabetes Care.

[17] Battelino T., Danne T., Bergenstal R.M. (2019). Clinical targets for continuous glucose monitoring data interpretation: Recommendations from the international consensus on time in range.. Diabetes Care.

[18] Suzuki D., Yamada H., Yoshida M. (2020). Sodium–glucose cotransporter 2 inhibitors improved time‐in‐range without increasing hypoglycemia in Japanese patients with type 1 diabetes: A retrospective, single‐center, pilot study.. J. Diabetes Investig..

[19] Kaviarasan S., Muniandy S., Qvist R., Ismail I.S (2009). F(2)-isoprostanes as novel biomarkers for type 2 diabetes: A review.. J. Clin. Biochem. Nutr..

[20] Service F.J. (2013). Glucose variability.. Diabetes.

[21] Jin S.M., Kim T.H., Bae J.C. (2014). Clinical factors associated with absolute and relative measures of glycemic variability determined by continuous glucose monitoring: An analysis of 480 subjects.. Diabetes Res. Clin. Pract..

[22] Yoo S., Chin S.O., Lee S.A., Koh G. (2015). Factors associated with glycemic variability in patients with type 2 diabetes: Focus on oral hypoglycemic agents and cardiovascular risk factors.. Endocrinol. Metab..

[23] Chiba K., Nomoto H., Nakamura A., Cho K.Y., Yamashita K., Shibayama Y. (2020). Sodium–glucose cotransporter 2 inhibitors reduce day-to-day glucose variability in patients with type 1 diabetes.. J. Diabetes Investig..

[24] Nathan D.M., Kuenen J., Borg R., Zheng H., Schoenfeld D., Heine R.J. (2008). Translating the A1C assay into estimated average glucose values.. Diabetes Care.

[25] Vigersky R.A., McMahon C. (2019). The relationship of hemoglobin a1c to time-in-range in patients with diabetes.. Diabetes Technol. Ther..

[26] Borg R., Kuenen J.C., Carstensen B. (2010). Associations between features of glucose exposure and A1C: the A1C-Derived Average Glucose (ADAG) study.. Diabetes.

[27] Vasilakou D., Karagiannis T., Athanasiadou E., Mainou M., Liakos A., Bekiari E. (2013). Sodium-glucose cotransporter 2 inhibitors for type 2 diabetes: A systematic review and meta-analysis.. Ann. Intern. Med..

[28] Shao S.C., Chang K.C., Lin S.J. (2020). Favorable pleiotropic effects of sodium glucose cotransporter 2 inhibitors: Head-to-head comparisons with dipeptidyl peptidase-4 inhibitors in type 2 diabetes patients.. Cardiovasc. Diabetol..

[29] Taylor P.J., Lange K., Thompson C.H., Gary W., Brinkworth G.D. (2018). Association of glycemic variability and the anti-glycemic medication effect score in adults with type 2 diabetes.. Diabetes Manag..

[30] Kohnert K., Heinke P., Vogt L., Zander E., Fritzsche G., Augstein P. (2011). Reduced glucose variability is associated with improved quality of glycemic control in patients with type 2 diabetes: A 12-month observational study.. J. Endocrinol. Metab..

